# On the taxonomy of the genus *Sacada* Walker, 1862 from India, with descriptions of a new genus and two new species (Pyralinae, Pyralidae, Lepidoptera)

**DOI:** 10.3897/zookeys.962.51194

**Published:** 2020-08-20

**Authors:** Navneet Singh, Jagbir Singh Kirti, Rahul Ranjan, Kailash Chandra, Wolfgang Speidel

**Affiliations:** 1 Zoological Survey of India, M–Block New, Alipore, Kolkata 700 053, West Bengal, India Zoological Survey of India Alipore India; 2 Department of Zoology and Environmental Sciences, Punjabi University, Patiala 147 002, Punjab, India Punjabi University Patiala India; 3 Museum Witt, Tengster, 33, 80796, München, Germany Museum Witt München Germany

**Keywords:** distribution, *Pseudosacada* gen. nov., *Sacada dzonguensis* sp. nov., *S. umtasorensis* sp. nov., taxonomic key, world checklist

## Abstract

Two new species, *Sacada dzonguensis* N. Singh, Kirti & Ranjan, **sp. nov.** and *S. umtasorensis* N. Singh, Kirti & Ranjan, **sp. nov.**, are described from India. Additionally, seven species of the genus *Sacada* Walker, 1862 are redescribed. A new genus, *Pseudosacada* N. Singh, Kirti & Ranjan, **gen. nov.**, is described to accommodate *Paravetta flexuosa* Snellen, 1890 (presently in *Sacada*). A new combination is established: *Pseudosacada flexuosa* (Snellen, 1890), **comb. nov.** Morphologically, the new genus resembles the genus *Sacada* and can only be diagnosed by the male genitalia. The diagnostic differences are discussed and illustrated along with adults and external male genitalia of related taxa. A world checklist and a key to the Oriental and Australasian species are provided.

## Introduction

The genus *Sacada* Walker, 1862 is a member of the family Pyralidae Latreille, 1809 and subfamily Pyralinae Latreille, 1809. It was established by monotypy for *S. decora* Walker, 1862 from Sarawak, Borneo. Hampson (1896) broadly discussed the nomenclature of this genus, synonymised several genera (i.e. *Sybrida* Walker, 1865, *Paravetta* Moore, 1865, *Danaka* Moore, 1879, and *Xestula* Snellen, 1885) with *Sacada* and studied nine species, which he divided into two distinct sections on the basis of male antennal characters: one group with bipectinate antennae with long branches along three-quarters of their length, and the other group with antennae serrate and fasciculate. Recently, Leraut (2013) revised the generic diagnosis of *Sacada* by including external genital attributes. The genus is known by 41 species, including 22 from the Oriental region and 10 from India (Nuss et al. 2003–2020).

Herein, two new species are described from India: *Sacada dzonguensis* N. Singh, Kirti & Ranjan, sp. nov. (Sikkim) and *S. umtasorensis* N. Singh, Kirti & Ranjan, sp. nov. (Meghalaya). In addition, the morphotaxonomy of seven Indian species of *Sacada* Walker, 1862 is studied. A new genus, *Pseudosacada* N. Singh, Kirti & Ranjan, gen. nov., is erected to accommodate *Paravetta flexuosa* Snellen, 1890 (presently in *Sacada*), and a new combination is established: *Pseudosacada flexuosa* (Snellen, 1890), comb. nov. Morphologically, the new genus resembles species of *Sacada* and can only be diagnosed by the male genitalia. The diagnostic differences are discussed and illustrated along with adults and external male genitalia of related taxa. A world checklist and identification key to the Oriental (23 species) and Australasian (four species) species are also provided. The distribution of species is updated from the publications by Hampson (1896), Yamanaka (1995, 1998), Nuss et al. (2003–2020), Bae et al. (2008), and Sutton et al. (2015) .

## Material and methods

Adult moths were collected using vertical sheet light traps fitted at various localities of India. Collected specimens were euthanized with ethyl acetate vapours in killing jars. The specimens were pinned, stretched, and processed as per standard techniques in lepidopterology. Adult moths were photographed using a Canon EOS 1300D digital SLR camera. The detailed microphotography of external male genitalia was performed under a Leica M165C stereomicroscope attached with a Leica MC190HD camera enabled with a Leica Application Suite. The examined specimens are deposited in the National Zoological Collections, Lepidoptera Section, Zoological Survey of India (ZSI), Kolkata, India.

**Abbreviations**:

**BMNH**Natural History Museum, London, UK (formerly the British Museum of Natural History)

**CMNH** Carnegie Museum of Natural History, Pittsburgh, Pennsylvania, USA

**HT** Holotype

**MGAB** Museum of Natural History "Grigore Antipa", Bucharest, Romania

**MNHN** Muséum National d’Histoire Naturelle, Paris, France

**MWNH** Museum Wiesbaden, Wiesbaden, Germany

**NHMUK** Natural History Museum, London, UK

**NZCZSI** National Zoological Collections, Zoological Survey of India, Kolkata, India

**OUMNH** Oxford University Museum of Natural History, Oxford, UK

**PT** Paratype

**RBINS** Royal Belgian Institute of Natural Sciences, Brussels, Belgium

**RMCA** Musée Royal de l’Afrique Centrale, Tervuren, Belgium

**RMNH** Naturalis Biodiversity Centre [formerly Rijksmuseum van Natuurlijke Historie], Leiden, the Netherlands

**TD** Type deposited

**TL** Type locality

**ZMHB** Museum für Naturkunde der Humboldt-Universität, Berlin, Germany

The collection abbreviations are according to Evenhuis (2020).

## Taxonomy

### Genus Sacada

Taxon classificationAnimaliaLepidopteraPyralidae

Walker, 1862

65F7FAF5-6F98-5B0B-942E-5AAA276D76B7

Sacada Walker 1862: 136.

#### Type species.

*Sacada decora* Walker, 1862.

#### Diagnostic characters.

Mostly dark-coloured moths with a slightly variable wing pattern; male antennae typically pectinate (ciliate and toothed in some species). In addition to the narrow forewing with angular edge and the sexual dimorphism with the female being much larger than the male, the genus *Sacada* is well defined by a number of characters: long legs with tufts of scales, some of which are filiform; thorax with patagia having prominent scales, ending with two brushes; male genitalia with uncus hooded; free valves without process; transtilla modified into elaborate sclerotized structure; juxta well developed; female genitalia with wide anal papillae; very short eighth segment; very short ductus bursae prolonged by a long, ovoid corpus bursae with sclerotisations (Leraut 2013).

#### Distribution.

Cameroon, China, Democratic Republic of the Congo, India, Indonesia, Ivory Coast, Japan, Madagascar, Malawi, Malaysia, Nigeria, Papua New Guinea, Russia, Uganda, Vietnam, Zimbabwe (Nuss et al. 2003–2020); Bhutan, Myanmar, Sri Lanka (Hampson 1896); Nepal (Yamanaka 1995).

##### Checklist of the genus *Sacada*

**Genus *Sacada* Walker, 1862**

=*Danaka* Moore, 1879

=*Datanoides* Butler, 1878

=*Kawiella* Roepke, 1943

=*Marionana* Viette, 1953

=*Paravetta* Moore, 1865

=*Sybrida* Walker, 1865

=*Xestula* Snellen, 1885

1 *Sacada acutipennis* (Strand, 1915) (*Aiteta*)

**TL.** Cameroon, Bang Manenguba Mountains

**TD.** ZMHB

**Distribution.** Cameroon (Bang Manenguba Mountains)

2 *Sacada albioculalis* Hampson, 1917

**TL.** Indonesia, New Guinea, West Papua [Dutch New Guinea], Fak-fak

**TD.** NHMUK

**Distribution.** Indonesia (New Guinea, West Papua, Fak-fak)

3 *Sacada amoyalis* Caradja, 1932

**TL.** China, Fujian, Xiamen [Amoy]

**TD.** MGAB

**Distribution.** China (Fujian, Xiamen [Amoy])

4 *Sacada approximans* (Leech, 1888) (*Datanoides*)

**TL.** Japan, Yokohama

**TD.** NHMUK

**Distribution.** Japan (Yokohama), Vietnam (Tam Ðảo, Vinh Phuc), Korea

5 *Sacada confutsealis* Caradja, 1925

**TL.** China, Fujian, Xiamen [Amoy]

**TD.** MGAB

**Distribution.** China (Fujian, Xiamen [Amoy])

6 *Sacada constrictalis* (Ragonot, 1891) (*Sybrida*)

**TL.** India, Upper Assam [Haut-Assam]

**TD.** ZMHB

**Distribution.** India (Upper Assam), Borneo

7 *Sacada contigua* South in Leech & South, 1901

**TL.** China, Pu-tsu-fong; Sichuan, Baoxing [Moupin]

**TD.** NHMUK

**Distribution.** China (Pu–tsu–fong, Sichuan)

8 *Sacada decora* Walker, 1862

**TL.** Malaysia, Borneo, Sarawak

**TD.** OUMNH

**Distribution.** India. Uttarakhand (Kumaon, Dehradun), Sikkim, Nagaland (Chizami), China (Yunnan), Myanmar, Nepal, Thailand, Vietnam, Malaysia (Borneo, Sarawak).

9 *Sacada dipenthes* Meyrick, 1934

**TL.** DR Congo [Belgian Congo], Lubumbashi [Elisabethville]

**TD.** RMCA

**Distribution.** DR Congo (Lubumbashi [Elisabethville])

10 *Sacada discinota* (Moore, 1865 [66]) (*Paravetta*)

**TL.** India, West Bengal, Darjeeling

**TD.** NHMUK

**Distribution.** India (West Bengal, Darjeeling), Nepal

11 *Sacada dzonguensis* N. Singh, Kirti & Ranjan, sp. nov.

**TL.** India, Sikkim, Dzongu

**TD.**NZCZSI

**Distribution.** India (Sikkim)

12 *Sacada erythropis* Hampson, 1917

**TL.** S. [West] Nigeria, Kwara, Ilorin

**TD.** NHMUK

**Distribution.** S. [West] Nigeria (Kwara, Ilorin)

13 *Sacada fasciata* (Butler, 1878) (*Datanoides*)

=*Xestula miraculosa* Snellen, 1885; **TL.** Russia, Amur river area [pays de la rivière Amour] **TD.**NHMUK; Distribution. Russia (Amur)

**TL.** Japan, Yokohama

**TD.** NHMUK

**Distribution.** Japan (Yokohama), Russia (Amur), Korea

14 *Sacada giovanettae* (Marion, 1957) (*Danaka*)

**TL.** Ivory Coast

**TD.** MNHN

**Distribution.** W. Africa (Ivory Coast)

15 *Sacada hoenei* Caradja & Meyrick, 1937

**TL.** China, Yülingshan

**TD.** MGAB

**Distribution.** China (Yunnan)

16 *Sacada inordinata* (Walker, 1865) (*Sybrida*)

**TL.** India, West Bengal, Darjeeling

**TD.** NHMUK

**Distribution.** India (West Bengal, Darjeeling)

17 *Sacada madegassalis* Viette, 1960

**TL.** Madagascar

**TD.** MNHN

**Distribution.** Madagascar

18 *Sacada metaxantha* Hampson, 1906

**TL.** Indonesia, New Guinea, West Papua, Kapaur

**TD.** NHMUK

**Distribution.** Indonesia (New Guinea, West Papua, Kapaur)

19 *Sacada misakiensis* (Shibuya, 1928) (*Sybrida*)

**TL.** Japan, Osaka, Misaki

**TD.** Not known

**Distribution.** Japan (Osaka, Misaki)

20 *Sacada nicopaea* Tams, 1941

**TL.** Uganda

**TD.** NHMUK

**Distribution.** Uganda (Kampala)

21 *Sacada nigripuncta* Hampson, 1906

**TL.** Indonesia, New Guinea, West Papua, Kapaur

**TD.** NHMUK

**Distribution.** Indonesia (New Guinea, West Papua, Kapaur)

22 *Sacada nyasana* Hampson, 1917

**TL.** Malawi [British Central Africa], Mt Mulanje

**TD.** NHMUK

**Distribution.** Malawi (Mt Mulanje)

23 *Sacada olivina* Joannis, 1930 [29]

**TL.** Tonkin [Vietnam], Hoang su phi

**TD.** MNHN

**Distribution.** Vietnam (Tonkin, Hoang su phi)

24 *Sacada pallescens* Hampson, 1896

**TL.** India, Sikhim, [Sikkim]

**TD.** NHMUK

**Distribution.** India (Sikkim), Bhutan, Vietnam, Nepal

25 *Sacada papuana* Hampson, 1917

**TL.** Papua New Guinea [British New Guinea], Dinawa

**TD.** NHMUK

**Distribution.** Papua New Guinea (Dinawa)

26 *Sacada paraxantha* Meyrick, 1936

**TL.** Democratic Republic of the Congo [Belgian Congo], Lubumbashi [Elisabethville]

**TD.** RMCA

**Distribution.** Democratic Republic of the Congo (Lubumbashi)

27 *Sacada paulianalis* (Viette, 1953) (*Marionana*)

=*Marionana vinolentalis* Viette, 1960; **TL.** Madagascar, Route ďAnosibé; **TD.**MNHN; **Distribution.** Madagascar

**TL.** Madagascar, Périnet, forêt du domaine de l’Est

**TD.** MNHN

**Distribution.** Madagascar

28 *Sacada peltobathra* Meyrick, 1938

**TL.** Indonesia, Java, Mt Guntur

**TD.** NHMUK

**Distribution.** Indonesia (Sumatra, Java. Mt Guntur)

29 *Sacada pusilla* Hering, 1901

**TL.** Indonesia, Sumatra

**TD.** Not known

**Distribution.** Indonesia (Sumatra)

30 *Sacada pyraliformis* (Moore, 1879) (*Danaka*)

**TL.** India, West Bengal, Darjiling

**TD.** ZMHB

**Distribution.** India (West Bengal, Darjeeling), Nepal, Myanmar, Thailand

31 *Sacada ragonotalis* (Snellen, 1892) (*Sybrida*)

= *Kawiella testacea* Roepke, 1943; **TL.** Indonesia, W Java, Perbawattee **TD.**RMNH; **Distribution.** Indonesia (Java)

**TL.** Indonesia, Java

**TD.** Syntypes in MWNH

**Distribution.** Indonesia (Sumatra, Java, Bali), Borneo

32 *Sacada rhodinalis* Hampson, 1906

**TL.** Zimbabwe, Mashonaland

**TD.** NHMUK

**Distribution.** Zimbabwe (Mashonaland)

33 *Sacada rhyacophila* (Ghesquière, 1942) (*Danaka*)

**TL.** DR of the Congo [Congo belge], Equateur, Bolombo

**TD.** RMCA

**Distribution.** Democratic Republic of the Congo

34 *Sacada rosealis* Hampson, 1906

**TL.** Zimbabwe [Mashonaland], Harare [Salisbury]

**TD.** NHMUK

**Distribution.** Zimbabwe (Mashonaland, Harare)

35 *Sacada rubralis* Holland, 1900

**TL.** Indonesia, Maluku, Buru

**TD.** CMNH

**Distribution.** Indonesia (Maluku, Buru)

36 *Sacada rufina* Hampson, 1896

**TL.** India, Maharashtra, Mumbai [Bombay]

**TD.** NHMUK

**Distribution.** India (Maharashtra, Mumbai [Bombay])

37 *Sacada sikkima* (Moore, 1879) (*Paravetta*)

**TL.** India, West Bengal, Darjeeling

**TD.** Syntype in NHMUK

**Distribution.** India (West Bengal, Darjeeling), Nepal

38 *Sacada szetschwanalis* Caradja, 1927

**TL.** China, Sichuan (Kwanhsien Talbo)

**TD.** MGAB

**Distribution.** China (Sichuan)

39 *Sacada tonsealis* Roepke, 1938

**TL.** Indonesia, northern Sulawesi

**TD.** RBINS

**Distribution.** Indonesia (North Celebes [Sulawesi]), Borneo

40 *Sacada umtasorensis* N. Singh, Kirti & Ranjan, sp. nov.

**TL.** India, Meghalaya, Umtasor

**TD.**NZCZSI

**Distribution.** India (Meghalaya)

41 *Sacada unilinealis* Hampson, 1896

**TL.** India, Sikhim [Sikkim]

**TD.** NHMUK

**Distribution.** India (Sikkim)

42 *Sacada viridalis* Hampson, 1917

**TL.** Cameroon, Ja R[iver], Bitje

**TD.** NHMUK

**Distribution.** Cameroon

### Sacada sikkima

Taxon classificationAnimaliaLepidopteraPyralidae

(Moore, 1879)

4CF4E438-FA00-57C4-BB60-D7EF063251E9

**Figures 1–6.**
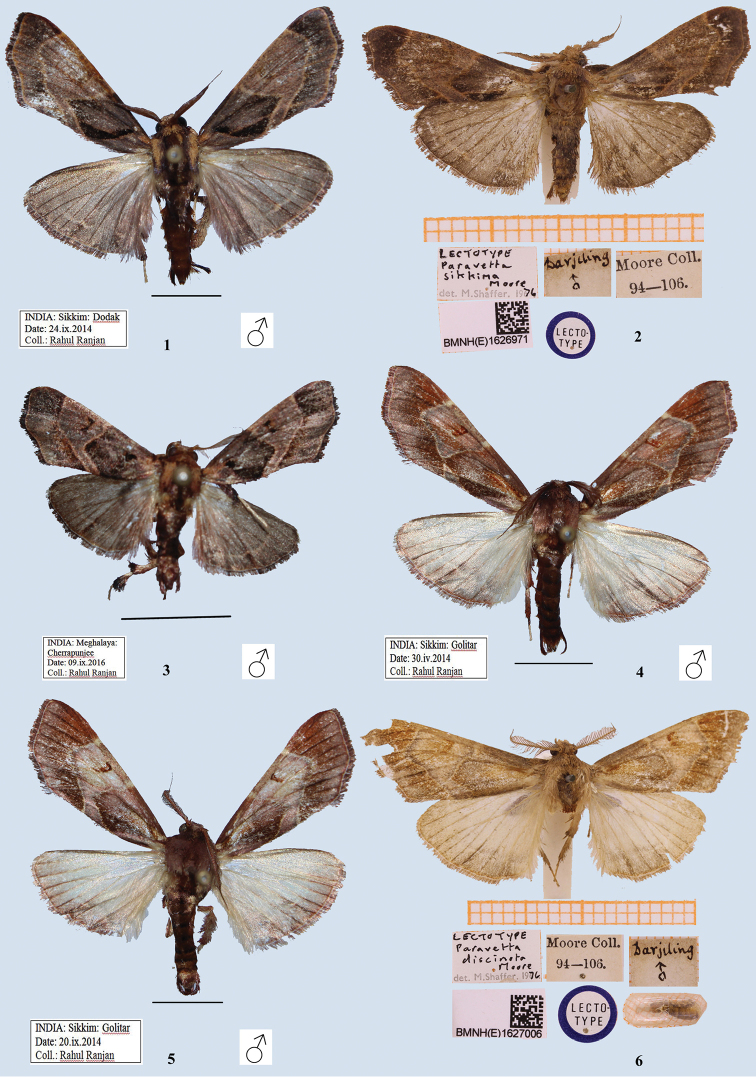
Adults of *Sacada* spp. **1***S. sikkima* (Moore) (male), India **2***S. sikkima* (Moore) (male), lectotype, Darjeeling, India **3***S. constrictalis* (Ragonot) (male), India **4, 5***S. discinota* (Moore) (male), India **6***S. discinota* (Moore) (male), lectotype, Darjeeling, India. Scale bars: 5 mm (**1**); 12.7 mm (**3–5**).

Figs 1 , 2 , 19 , 20

Paravetta sikkima Moore 1879: 70.

#### Description.

Male, wingspan 28 mm (Figs 1, 2). Adult dark purplish fuscous. Forewing with a dark rufous rectangular patch near base, touching antemedial line which is highly angled in interno-median interspace; postmedial line pale, sinuous, outwardly oblique from costa to vein M_2_, then very oblique to inner margin; area between antemedial and postmedial line paler and beyond postmedial line darker. Hindwing pale brown; a pale, slightly waved submarginal line crossed by a dark streak at vein Cu_1_. *Male genitalia* (Figs 19, 20). Uncus broad with flaps on lateral side, gnathos reaching up to tip of uncus, tip hooked; valva simple, without any process; tegumen simple; transtilla broad with sclerotised, bifid process originating medially; juxta in form of two long arms, broad medially, spined apically; saccus deeply U-shaped; vesica membranous with fine scobination, without any cornuti.

#### Diagnosis.

*Sacada sikkima* is externally similar to *S. constrictalis* from India, but differs by its larger size, and in having the postmedial line outwardly oblique from the costa to vein M_2_, whereas, in *S. constrictalis* the postmedial lines is almost straight. In the male genitalia (Figs 19, 20), the transtillar processes are longer; the juxta is larger.

#### Type material examined.

Lectotype (Fig. 2): BMNH (E) 1626971, male, Darjeeling, Moore coll. 94–106, *Paravetta sikkima* Moore, det. M. Shaffer, 1976.

#### Other material examined.

India, Sikkim: 1 ♂, Dodak, 24.ix.2014, leg. R. Ranjan (Coll. NZCZSI). India, Uttarakhand: 1 ♂, Dehradun, 22.v.2014, leg. R. Ranjan (Coll. NZCZSI). India, Meghalaya: 1 ♂, Umtasor, 15.ix.2014, leg. R. Ranjan (Coll. NZCZSI). India, Mizoram: 1 ♂, Mamit, 08.ix.2016, leg. R. Ranjan (Coll. NZCZSI); India, Arunachal Pradesh: 1 ♂, Dibang valley, Italin, 26.x.2017, leg. R. Ranjan (Coll. NZCZSI).

### Sacada constrictalis

Taxon classificationAnimaliaLepidopteraPyralidae

(Ragonot, 1891)

3FEA9E56-789E-5C4C-BD4D-AA7F7ECE4E37

Figs 3 , 21 , 22

Sybrida constrictalis Ragonot 1891: 75–76, pl. 8 fig. 10.

#### Description.

**Male**, wingspan 24 mm (Fig. 3). Adult dark purplish fuscous. Forewing with a dark rufous rectangular patch near base, touching antemedial line, which is highly angled in interno-median interspace; postmedial line pale, sinuous, nearly orthogonal from costa to vein M_2_, then very oblique to inner margin; area between antemedial and postmedial line paler; discocellular with two specks, outer one darker. Hindwing pale fuscous, submarginal line pale, slightly waved, crossed by a dark streak at vein Cu_1_. Cilia of both wings ochreous, with two black lines passing through them. ***Male genitalia*** (Figs 21, 22). Uncus broad with flaps on lateral side; gnathos with tip hooked; valva simple, without any process; tegumen simple; transtilla broad and sclerotised, bifid process originating medially; juxta broad with a vertical incision from tip to base, forming two arms, spined apically; saccus U-shaped; vesica membranous with fine scobination, without any cornuti.

#### Diagnosis.

Provided with the diagnosis of *S. sikkima.*

#### Material examined.

India, Meghalaya: 3 ♂, Cherrapunji, 04.ix.2014, leg. R. Ranjan (Coll. NZCZSI); 1 ♂, Umtasor, 15.ix.2014, leg. R. Ranjan (Coll. NZCZSI).

### Sacada discinota

Taxon classificationAnimaliaLepidopteraPyralidae

(Moore, 1865)

6D0E653B-DA57-57B1-AFFF-BAEB265FF351

Figs 4–6 , 23 , 24

Paravetta discinota Moore 1865: 814, pl. 43 fig. 3.

#### Description.

**Male**, wingspan 32 mm (Figs 4–6). Forewing pale brown, a pale antemedial line, acutely angled in interno-median interspace with fuscous brown rectangular patch on its inner area and a similar postmedial line acutely angled at vein M_1_ (in one Golitar (Sikkim) specimen, angled antemedial line touches postmedial line at vein Cu_2_; Fig. 4); area between two lines pale brown with oblique ferruginous reniform spot. Hindwing pale; traces of a waved submarginal line; underside paler with similar markings. Thorax with long, brown patagia. ***Male genitalia*** (Figs 23, 24). Uncus broad, laterally folded, apically rounded; gnathos short and well developed, reaching up to midst of uncus, tip hooked; valva simple, without any process; tegumen broad; transtilla broad, a sclerotised flap-like process originating medially; juxta long, broad, slightly constricted at apex; vinculum U-shaped; aedeagus long, sclerotized carinal plate with numerous spikes; vesica membranous with fine scobination, cornuti absent.

#### Diagnosis.

Among the *Sacada* species reported from India, *S. discinota* is externally similar to *S. sikkima* and *S. constrictalis* due to the highly angled antemedial and postmedial lines, but it is distinct from both of these congeners by its paler hindwings.

#### Type material examined.

Lectotype (Fig. 6): BMNH (E) 1627006, male, Darjeeling, Moore Coll. 94–106, *Paravetta discinota* Moore, det. M. Shaffer, 1976.

#### Other material examined.

India, Sikkim: 4 ♂, Golitar, 20.ix.2014, leg. R. Ranjan (Coll. NZCZSI); 1 ♂, Dodak, 24.ix.2014; 6 ♂, Golitar, 30.iv.2014, leg. R. Ranjan (Coll. NZCZSI); 3 ♂, Golitar, 19.ix.2014, leg. R. Ranjan (Coll. NZCZSI); 1 ♂, Chungthang, 26.iv.2014, leg. R. Ranjan (Coll. NZCZSI).

#### Remark.

The lectotype is hereby formally designated.

### Sacada unilinealis

Taxon classificationAnimaliaLepidopteraPyralidae

Hampson, 1896

78A33B09-D1C7-59EE-8EAF-0B636159D9C1

**Figures 7–12.**
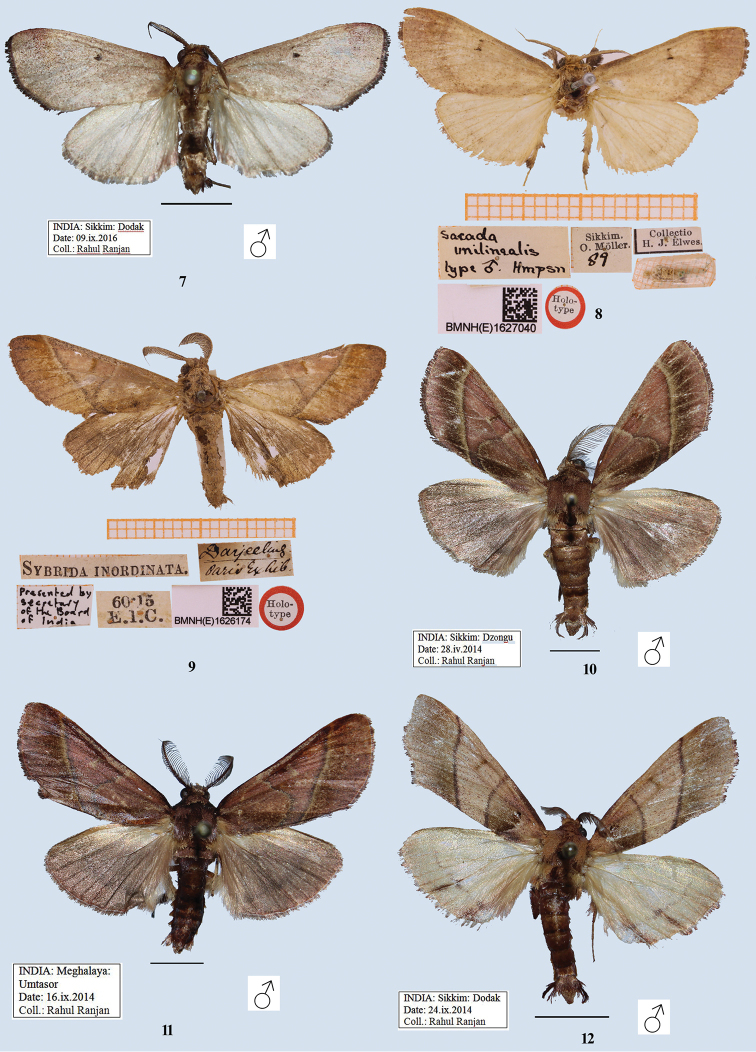
Adults of *Sacada* spp. **7***S. unilinealis* Hampson (male), India **8***S. unilinealis* Hampson (male), holotype, Sikkim, India **9***S. inordinata* (Walker) (male), holotype, Darjeeling, India **10***S. dzonguensis*, sp. nov. (male), India. **11***S. umtasorensis*, sp. nov. (male), India **12***S. pallescens* Hampson (male), India. Scale bars: 5 mm (**7, 10, 11**); 12.7 mm (**12**).

Figs 7 , 8 , 25 , 26

Sacada unilinealis Hampson 1896: 170.

#### Description.

**Male**, wingspan 32–34 mm (Figs 7, 8). Adult pale rufous, speckled with fuscous; forewing pale brownish pink; basal and apical area of costa rufous; forewing with two black specks (lower one large, giving appearance of a spot) conjoined by a narrow bar; traces of evenly curved postmedial line, with area beyond it darker. Hindwing pale, with faint traces of a curved submarginal line. Cilia of both wings dark rufous. Blackish fringe of hair on fore and mid tibiae. ***Male genitalia*** (Figs 25, 26) with uncus short, broad with flaps on lateral side; gnathos well developed reaching to uncus, tip hooked; valva broad, simple, without any process; tegumen simple; transtilla with a sclerotised process arising medially; juxta double, each broad at base, apically pointed and sclerotised, concave on inner edge, convex on outer edge; saccus long, broadly U-shaped; vesica membranous with fine scobination, without any cornuti.

#### Diagnosis.

*Sacada unilinealis* is an unmistakable species due to the weak markings and almost uniform colour of the fore and hindwings.

#### Type material examined.

Holotype (Fig. 8): BMNH (E) 1627040, male, Sikkim, O. Möller, 89, collection H. J. Elwes, *Sacada unilinealis* Hampson.

#### Other material examined.

India, Sikkim: 1 ♂, Dodak, 09.ix.2016, leg. R. Ranjan (Coll. NZCZSI)

### Sacada inordinata

Taxon classificationAnimaliaLepidopteraPyralidae

(Walker, 1865)

7225F440-CD05-5975-A020-D0927E88C2E1

Fig. 9

Sybrida inordinata Walker 1865: 466.

#### Description.

Adults are rufous. Forewing with diffused a ferruginous patch in interno-median interspace; a medial line approximately right angled, reaching at vein Cu_2_; postmedial line obliquely straight with some ferruginous beyond it, merged the medial line at Cu_2_ and touching the inner margin; a ferruginous line on discocellular; termen smoothly curved. Hindwing browner, with traces of dark postmedial line.

#### Diagnosis.

Provided with the following species.

#### Type material examined.

Holotype, male, BMNH (E) 1626174, *Sybrida inordinata*, Darjeeling, 60-15 E. I. C. [East India Company].

### Sacada dzonguensis

Taxon classificationAnimaliaLepidopteraPyralidae

N. Singh, Kirti & Ranjan sp. nov.

81DE7752-5D58-5A75-87EA-AA4154A58F40

http://zoobank.org/E2147930-463E-4DF6-ABD3-A500CC3FFA88

Figs 10 , 27 , 28

#### Description.

**Male**, wingspan 36 mm (Fig. 10). Rufous brown. Forewing with a medial fuscous line outwardly oblique from costa to vein Cu_2_, slightly indented in cell, at Cu_2_ rounded inwardly to meet inner margin; a dark streak on discocellular; a postmedial fuscous line, inwardly oblique from radial veins; inner area of antemedial and outer area of postmedial lines bordered with ochreous scales; a broad fuscous band beyond postmedial line, veins on it paler; inner area dark brownish; a fine marginal line, cilia brownish; underside rufous with inner area ochreous. Hindwing pale fuscous with rufous tinge; traces of diffuse, postmedial fuscous line; a fine marginal line present; underside rufous. ***Male genitalia*** (Figs 27, 28): uncus hooded with baso-lateral flaps; gnathos curved distally, tip pointed and hooked, broadened below tip; valva simple; transtilla broad and curved distally; juxta broad at base, mediolateral area constricted, bifid apically: both arms (spikes) bearing small spines; vinculum U-shaped; aedeagus apex with multiple rows of small spines; base of vesica densely scobinated and the scobination gradually becomes sparse towards distal end.

#### Diagnosis.

*Sacada dzonguensis* sp. nov. is most similar to *S. inordinata* (Fig. 9), but the forewing has the antemedial and postmedial lines clearly separated, and there is a broad fuscous band beyond the postmedial line, whereas in *S. inordinata* both lines are fused from vein Cu_2_ to the inner margin, and the postmedial fuscous band is absent (but with traces of ferruginous).

#### Type material.

Holotype, male. India, Sikkim: Dzongu, 28.iv.2014, leg. R. Ranjan (Coll. NZCZSI).

#### Etymology.

The species is named after its type locality, Dzongu, Sikkim, India.

### Sacada umtasorensis

Taxon classificationAnimaliaLepidopteraPyralidae

N. Singh, Kirti & Ranjan sp. nov.

0F5D6089-1C47-5B48-9089-A7E0A44793CD

http://zoobank.org/AE3EC692-2759-4260-829C-C01F12F03392

Figs 11 , 29 , 30

#### Description.

**Male**, wingspan 30 mm (Fig. 11). Rufous brown. Forewing with a sinuous medial fuscous line outwardly oblique from costa to vein Cu_2_, then broadly and inwardly rounded to meet inner margin; a band of paler scales on discocellular; postmedial fuscous line, slightly curved, inwardly oblique from costa to inner margin; inner area of medial line and outer area of postmedial line bordered with ochreous scales; a broad ferruginous band beyond postmedial line; a fine marginal line, cilia brownish; underside rufous with inner area ochreous. Hindwing pale fuscous with rufous tinge; traces of diffused, postmedial fuscous line; a fine marginal line present; underside rufous. ***Male genitalia*** (Figs 29, 30): uncus hooded with baso-lateral flaps; gnathos curved distally, hooked, tip pointed, broadened before tip; valva simple; transtilla broad with two apical, small thumb-like processes; juxta narrow, mediolateral area constricted, bifid apically with both the arms bearing spikes; vinculum U-shaped; aedeagus apex with single row of small spines; base of vesica densely scobinated and the scobination gradually becomes sparse towards apex.

#### Diagnosis.

*Sacada umtasorensis* sp. nov., distributed in Meghalaya is most closely similar to its allopatric relative *S. dzonguensis* sp. nov., (distributed in Sikkim) (Fig. 10), but it is distinct by the oblique postmedial line from costa to inner margin, whereas in *S. dzonguensis*, the postmedial line is straight from the costa to the radial vein and then oblique to the inner margin. In the male genitalia of *S. umtasorensis* (Figs 29, 30), the juxta is narrow with the two apical lobes exhibiting more spines, and the aedeagus apex has a single row of small spines, whereas in *S. dzonguensis* (Figs 27, 28), the juxta is broad, the apical lobes have fewer spines, and the aedeagus apex exhibits multiple rows of small spines.

#### Type material.

***Holotype***, male. India, Meghalaya: Umtasor, 16.ix.2014, leg. R. Ranjan (Coll. NZCZSI).

***Paratypes*** (9 ♂), India, Meghalaya: 1 ♂, Umtasor, 15.ix.2014; 8 ♂, 16.ix.2014, leg. R. Ranjan (Coll. NZCZSI).

#### Etymology.

The species is named after its type locality Umtasor, Meghalaya, India.

### Sacada pallescens

Taxon classificationAnimaliaLepidopteraPyralidae

Hampson, 1896

B02A278C-DC0A-53D2-9E31-DA712ABDA793

**Figures 13–18.**
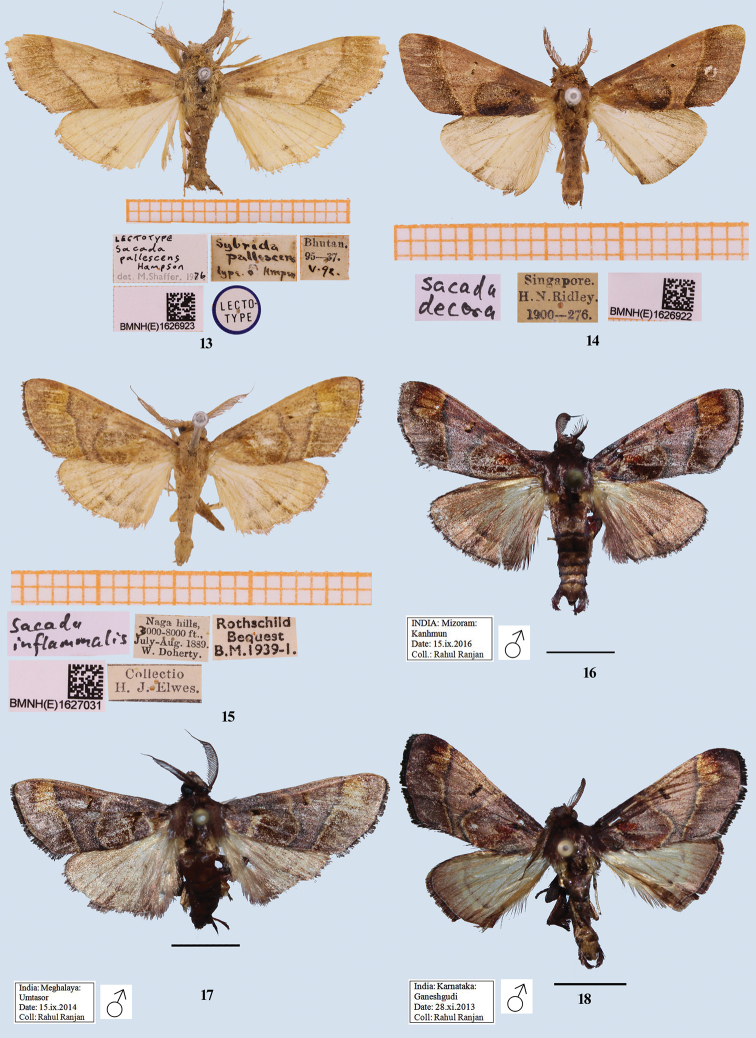
Adults of *Sacada* and *Pseudosacada* spp. **13***S. pallescens* Hampson (male), lectotype, Bhutan **14***S. decora* Walker, Singapore **15***Pseudosacada flexuosa* (Snellen) (= *Sybrida inflammealis* Ragonot), India **16***P. flexuosa* (Snellen) (male), Kanhmun, Mizoram, India **17***P. flexuosa* (Snellen) (male), Umtasor, Meghalaya, India **18***P. flexuosa* (Snellen) (male), Ganeshgudi, Karnataka, India. Scale bars: 5 mm (**16–18**).

Figs 12 , 13 , 31 , 32

Sacada pallescens Hampson 1896: 171.

#### Description.

**Male**, wingspan 32 mm (Figs 12, 13). Pale rufous. Forewing speckled fuscous; a dark brownish basal spot; antemedial line smoothly curved; a speck on discocellular; postmedial line slightly curved below costa, then oblique to inner margin, some fuscous suffusion beyond it; cilia dark at tips; underside ochreous with rufous suffusion on basal half of costa, curved postmedial line present. Hindwing pale with indistinct, evenly curved postmedial line, crossed by a rufous streak on vein Cu_2_. Underside with curved postmedial line. ***Male genitalia*** (Figs 31, 32). Uncus broad with a fold on lateral side; gnathos well developed, tip hooked; valva simple, without any process; tegumen broad; transtilla broad, forming inverted omega (ω) shape; juxta short and broad, slightly constricted at apex; saccus long; vinculum U-shaped; aedeagus long, vesica membranous with fine scobination, cornuti absent.

#### Diagnosis.

*Sacada pallescens* is unmistakable among the species studied due to the smoothly curved antemedial line (highly angled in other Indian species, except in *S. unilinealis* where it is absent) and hindwing which has a prominent rufous streak on vein Cu_2_.

#### Type material examined.

Lectotype (Fig. 13): BMNH (E) 1626923, male, Bhutan. 95–37.v.96, *Sybrida pallescens* Hampson/*Sacada pallescens* Hampson det. M. Shaffer, 1976.

#### Other material examined.

India, Sikkim: 1 ♂, Dodak, 24.ix.2014, leg. R. Ranjan (Coll. NZCZSI); India, Arunachal Pradesh: 1 ♂, Dibang valley, Italin, 26.x.2017, leg. N. Singh (Coll. NZCZSI).

#### Remark.

The lectotype is hereby formally designated.

### Sacada decora

Taxon classificationAnimaliaLepidopteraPyralidae

Walker, 1862

B528CB3F-D5C1-5A6B-8566-E52AC4A26970

Fig. 14

Sacada decora Walker 1862: 136.

#### Description.

**Male**, wingspan 25.4 mm (Fig. 14). Rosy red; forewing with antemedial line outwardly oblique, broadly and inwardly rounded at vein Cu_2_ to meet inner margin, where a black patch is present towards its inner edge; two black discal spots; an inwardly oblique, paler postmedial line followed by a broad band of fuscous scales, which is diffusing towards termen. Hindwing paler, a diffused postmedial line present.

#### Diagnosis.

Because of the smoothly curved postmedial line (not strongly angled), *S. decora* is externally similar to *S. inordinata*, *S. dzonguensis*, *S. umtasorensis*, and *S. pallescens*, but it differs from three of these four species having its hindwing paler, and from *S. pallescens* in having the antemedial line outwardly oblique and broadly and inwardly rounded at vein Cu_2_.

#### Material examined.

Singapore: hand written slip *Sacada decora*/BMNH (E) 1626922/1900-276/ H. N. Ridley

### Genus Pseudosacada

Taxon classificationAnimaliaLepidopteraNymphalidae

N. Singh, Kirti & Ranjan gen. nov.

1E6FDE4C-97F2-5CDF-A49F-408446EBD330

http://zoobank.org/42924214-79C7-4293-8591-1E2781DA1D44

#### Type species.

*Paravetta flexuosa* Snellen, 1890.

#### Diagnosis.

The new genus is morphologically most similar to the genus *Sacada* and can only be diagnosed on the basis of external male genitalia. In male genitalia, the uncus is broader at base, apically bifid with a shallow constriction. There are two strongly sclerotised processes arising from the latero-medial region of the uncus. The gnathos is long, reaching beyond the uncus, and with its apex having a small hook. The valva is simple and membranous, without any process. The transtilla is broad and with both the edges bearing scorpion’s “pedipalp chela"-like sclerotised process. In *Sacada*, the uncus is hooded, lateral structures are simple, flap-like, and without any horn-like process; the gnathos is short and hardly reaches the hood of the uncus; the valva is thicker; and the transtilla is simple.

#### Remarks.

The type species of the new genus was originally placed in *Paravetta* (type species *Paravetta discinota* Moore, 1865). *Paravetta* is now a synonym of *Sacada*. However, *P. flexuosa* is generically distinct from *Sacada decora*, the type species of *Sacada*, and therefore a new genus is erected here.

#### Etymology.

The genus is named for its morphological resemblance to some species of *Sacada*. The gender is feminine.

#### Distribution.

North-eastern India (Meghalaya, Mizoram, Sikkim), southern India (Karnataka); Myanmar; Vietnam; Nepal.

### Pseudosacada flexuosa

Taxon classificationAnimaliaLepidopteraNymphalidae

(Snellen, 1890) comb. nov.

5DC03D29-5C51-5E1C-B88F-64ADCBC71AC3

Figs 15–18 , 33–40

Paravetta flexuosa Snellen 1890: 558.
= Sybrida inflammealisRagonot 1891: 75.

#### TD.

Lectotype in NHMUK.

#### Description.

Male, wingspan 30 mm (Figs 15–18). Adult dark chocolate brown with fuscous and purple tinge; antennae bipectinate up to one-third of the length, apically simple; abdomen pale brownish; anal tufts rather strong; forewing with sub-basal, oblique purple patch below cell; antemedial line outwardly oblique from costa to vein Cu_2_, then rounded inward to meet inner margin, a small indention present in cell; postmedial line inwardly oblique, former inwardly and later outwardly bordered with ochreous scales; area between both lines distinctly differently coloured then rest of wing, an elongate spot on discocellular; on outer side of postmedial line, a roughly rectangular ochreous golden patch present from sub-costa to vein R_5_, veins on it dark. Hindwing ochreous brown with a curved postmedial line; outer area darker; underside paler; cilia as ground colour with fuscous basally. Hind tibia with two pairs of unequal tibial spurs covered with dark rufous scales, tip of each spur covered with whitish scales, one separate bunch of long rufous scales present. *Male genitalia* (Figs 33–40) discussed under the diagnosis of genus.

#### Material examined.

India, Meghalaya: 6 ♂, Umtasor, 16.ix.2014, leg. Rahul Ranjan (Coll. NZCZSI); 1 ♂, Umtasor, 15.ix.2014, leg. Rahul Ranjan (Coll. NZCZSI); 1 ♂, Mawsynram, 28.viii.2014, leg. Rahul Ranjan (Coll. NZCZSI). India, Mizoram: 2 ♂, Kanhmun, 15.ix.2016, leg. Rahul Ranjan (Coll. NZCZSI). India, Karnataka: 3 ♂, Ganeshgudi, 28.xi.2013, leg. Rahul Ranjan (Coll. NZCZSI). Fig. 15, *Sacada inflamm*[*e*]*alis*/ Naga Hills, 3000–8000 ft., July–Aug. 1889, W. Doherty/Rothschild Bequest B.M. 1939-1/ BMNH (E) 1627031/ Collectio[n] H. J. Elwes.

**Figures 19–26.**
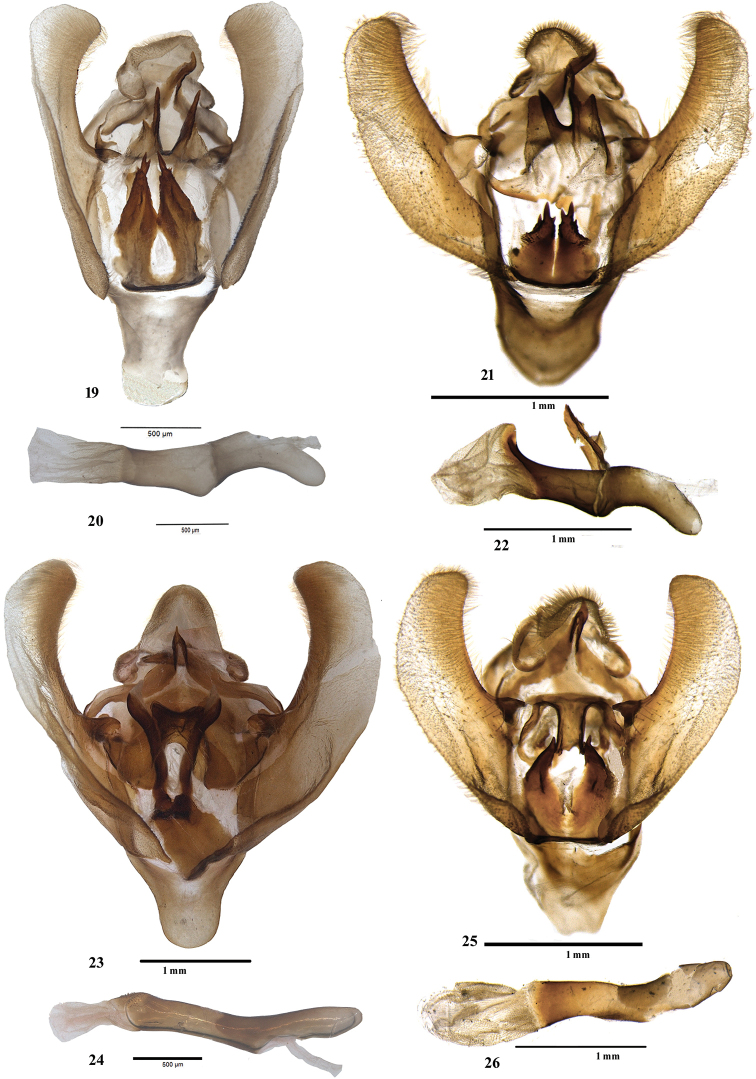
Male genitalia of *Sacada* spp. **19, 20** Male genitalia of *S. sikkima* (Moore) **21, 22** male genitalia of *S. constrictalis* (Ragonot) **23, 24** male genitalia of *S. discinota* (Moore) **25, 26** male genitalia of *S. unilinealis* Hampson.

#### Distribution.

North-eastern India (Sikkim, Meghalaya, Mizoram, Nagaland), southern India (Karnataka); Vietnam (Yên Bái); Nepal. Records of Mizoram and southern India are newly reported here.

**Figures 27–32.**
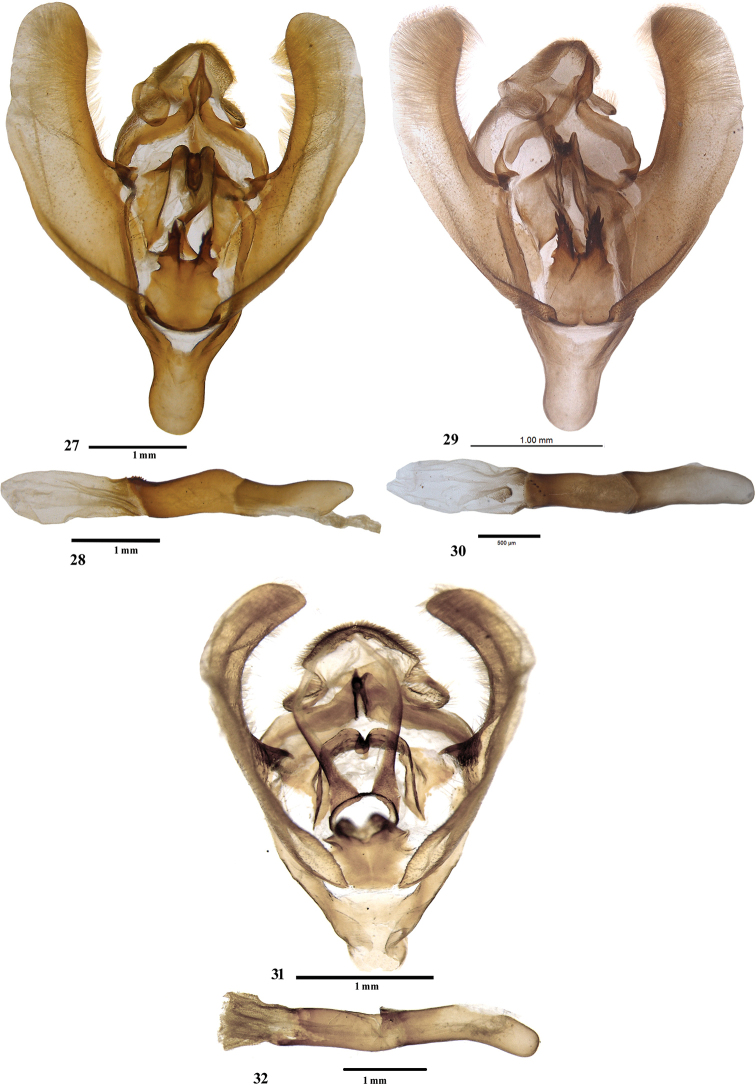
Male genitalia of *Sacada* spp. **27, 28** Male genitalia of *S. dzonguensis*, sp. nov. **29, 30** male genitalia of *S. umtasorensis*, sp. nov. **31, 32** male genitalia of *S. pallescens* Hampson.

**Figures 33–40.**
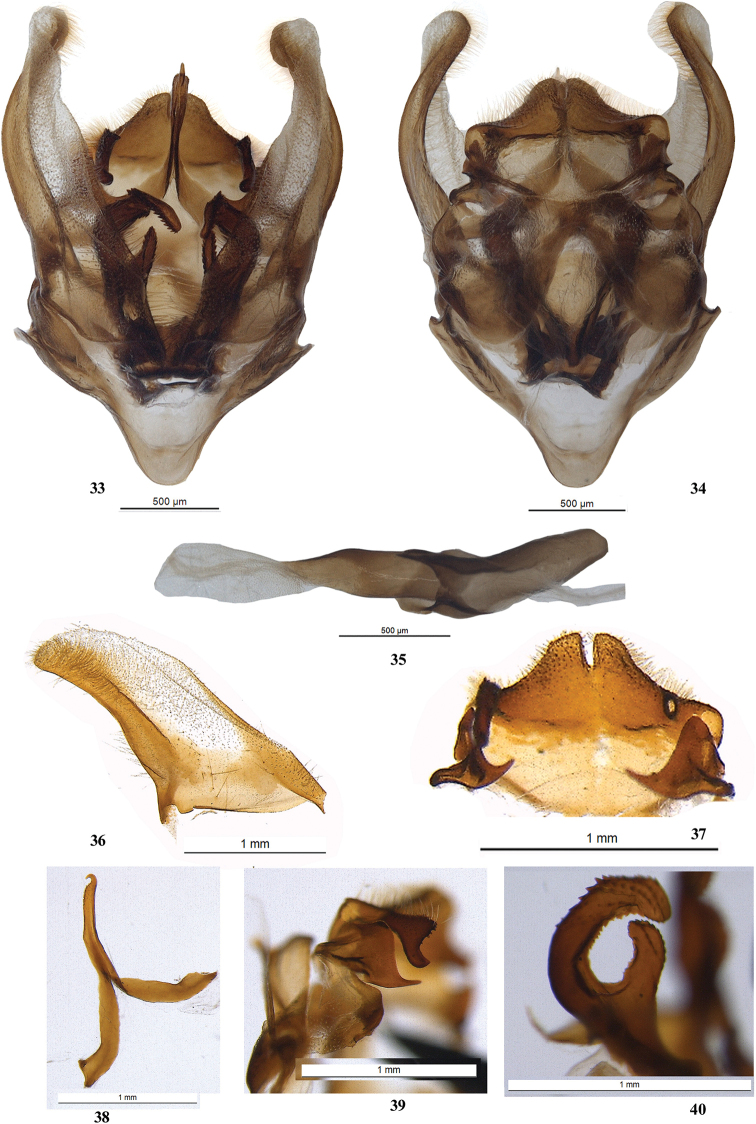
Male genitalia of *Pseudosacada flexuosa* (Snellen). **33** Ventral view **34** dorsal view **35** aedeagus **36** valva **37** uncus **38** gnathos **39** lateral process of uncus **40** enlarged view of transtilla processes.

### Identification key to the Oriental and Australasian species of *Sacada*

**Table:** 

1	Hindwing with smoky brown marginal band	**2**
–	Hindwing without any marginal band	**3**
2	Forewing with antemedial and medial lines well separated	***S. amoyalis***
–	Forewing with antemedial and medial lines merged with each other at inner area	***S. confutsealis***
3	Hindwing with postmedial/submarginal line	**10**
–	Hindwing without any postmedial/submarginal line	**4**
4	Forewing with dark spot or white line present	**5**
–	Forewing without any dark spot or white line	***S. metaxantha***
5	Forewing with antemedial and post medial line outlined	***S. ragonotalis***
–	Forewing with antemedial and postmedial line without any outline	**6**
6	Forewing with thin white line closing end of cell	***S. rubralis***
–	Forewing without fine white line at end of cell	**7**
7	Forewing with postmedial line strongly excurved at medial veins, then oblique to meet inner margin	***S. szetschwanalis***
–	Forewing with postmedial line not as above	**8**
8	Forewing with postmedial line approximately oblique	**9**
–	Forewing with postmedial line slightly wavy	***S. approximans***
9	Hindwing darker	***S. tonsealis***
–	Hindwing paler	***S. peltobathra***
10	Hindwing with postmedial/submarginal line incomplete	**11**
–	Hindwing with postmedial/submarginal line complete	**14**
11	Forewing expenses about 20 mm (± 2–3 mm)	**12**
–	Forewing expenses greater than 30 mm	**13**
12	Hindwing with three dark spots	***S. pusilla***
–	Hindwing without dark spots	***S. constrictalis***
13	Forewing with purplish rufous ground colour	***S. discinota***
–	Forewing with purplish fuscous ground colour	***S. sikkima***
14	Near the base of forewing a large transversely oblong whitish ringlet which encloses a black patch	***S. decora***
–	Forewing lacks the above attribute	**15**
15	Hindwing yellowish, redder towards outer margin	***S. rufina***
–	Hindwing not as above	**16**
16	Forewing with antemedial and postmedial line fused	**17**
–	Forewing with antemedial and postmedial line not fused	**18**
17	Forewing with antemedial and postmedial line fused from Cu_2_ to inner margin	***S. inordinata***
–	Forewing with antemedial and postmedial line fused at inner margin, forming V-shaped figure	***S. olivina***
18	Forewing with single speck	**19**
–	Forewing with two specks (separate or joined by a bar)	**21**
19	Hindwing with postmedial line crossed by a rufous streak on vein Cu_2_	***S. pallescens***
–	Hindwing without any streak on postmedial line	**20**
20	Forewing with an olive-green cell spot	***S. pyraliformis***
–	Forewing with a reddish brown discoidal spot defined by grey	***S. papuana***
21	Forewing without antemedial line, postmedial line present	***S. unilinealis***
–	Forewing with both the lines (antemedial and postmedial) present	**22**
22	Forewing with a large, fiery red or yellowish rufous patch below the cell before the antemedial line	**23**
–	Forewing without such patch below the cell before the antemedial line	**25**
23	Forewing with a large yellowish rufous patch below the cell before the antemedial line	***S. nigripuncta***
–	Forewing with a large fiery red patch below the cell before the antemedial line	**24**
24	Hindwing whitish, suffused with pale reddish	***S. albioculalis***
–	Hindwing fuscous; postmedial curved line whitish, area beyond it reddish brown	***S. hoenei***
25	Forewing with postmedial line highly angled	***S. contigua***
–	Forewing with postmedial line nearly oblique (not angled)	**26**
26	Forewing with postmedial line oblique from costa to inner margin	***S. umtasorensis* sp. nov.**
–	Forewing with postmedial line straight from costa to radial vein and then oblique to inner margin	***S. dzonguensis* sp. nov.**

## Discussion

After the description of two new *Sacada* species and the transfer of one species to *Pseudosacada* gen. nov., the genus *Sacada* now comprises 42 species worldwide, including 23 from the Oriental region and 11 from India. With 13 *Sacada* species, the Afrotropical region is the next most diverse region for this genus, and a future systematic revision should focus on these species. Apart from this, the Australasian region, with four species (included in the identification key) and the East Palaearctic region with two species (*S. fasciata*, *S. misakiensis*) need study to investigate the correct placement of *Sacada* from these regions based on features of genitalia morphology.

## Supplementary Material

XML Treatment for Sacada

XML Treatment for Sacada sikkima

XML Treatment for Sacada constrictalis

XML Treatment for Sacada discinota

XML Treatment for Sacada unilinealis

XML Treatment for Sacada inordinata

XML Treatment for Sacada dzonguensis

XML Treatment for Sacada umtasorensis

XML Treatment for Sacada pallescens

XML Treatment for Sacada decora

XML Treatment for Pseudosacada

XML Treatment for Pseudosacada flexuosa
